# Nursery Resource Use Dynamics in Great Hammerheads ( *Sphyrna mokarran* ) Across Ontogeny

**DOI:** 10.1002/ece3.71473

**Published:** 2025-06-16

**Authors:** John F. Hlavin, Catherine C. Macdonald

**Affiliations:** ^1^ Shark Research and Conservation Program University of Miami Rosenstiel School for Marine, Atmospheric, and Earth Science Key Biscayne Florida USA; ^2^ Field School Scientific Training Coconut Grove Florida USA

**Keywords:** diet, hammerhead, niche, nursery, ontogenetic shift, shark, stable isotope analysis

## Abstract

Dependence on nursery areas often requires young sharks to make trade‐offs between access to prey and avoiding the risk of predation, potentially resulting in constrained habitat and resource use that elevates their susceptibility to anthropogenic disturbance. We investigated trends in ontogenetic and seasonal resource use in Critically Endangered great hammerhead sharks ( *Sphyrna mokarran* ) during and after association with an urbanized nursery area in Biscayne Bay, FL, using analysis of carbon and nitrogen isotopes in muscle and plasma. We found that great hammerheads ranging from 63 to 287 cm fork length (FL) showed a partial ontogenetic shift from bay to coastal resources, predicted to occur around ~125 cm FL (or ~2 years old). Bayesian models suggested dependence on bay batoids supplemented by bay teleosts during nursery association, before transitioning to a coastal teleost‐dominated diet. Significantly larger isotopic niches and greater trophic diversity were observed among subadults and adults relative to juveniles, with some also incorporating coastal shark prey alongside teleosts and others continuing to primarily exploit bay prey groups. Subadults transitioning away from year‐round nursery dependence also exhibited clear seasonal variation in foraging, switching from coastal foraging during the dry season to bay foraging during the wet season, potentially due to a seasonal increase in the abundance of adult hammerheads in the area from the late dry to the early wet season. Overall, results suggest that the resource use of immature great hammerheads may be constrained by competition, predation, and specialization, potentially driving vulnerability to anthropogenic disturbance of critical nursery prey and habitats.

## Graphical abstract

The ecological trade‐offs required by nursery dependence are poorly understood. Using multi‐tissue stable isotope analysis, this research provides evidence of constrained resource use in juvenile great hammerheads ( *Sphyrna mokarran* ) associated with year‐round dependence on the Biscayne Bay nursery during their first two years of life. Subadult hammerheads shift to coastal foraging but seasonally increase bay foraging, potentially to reduce interactions with adult conspecifics which are more abundant in those months. Specialized use of bay habitats by immature individuals suggests elevated vulnerability to anthropogenic disturbance, while their expansion into coastal foraging habitats necessitates inclusion of those areas in nursery boundaries.

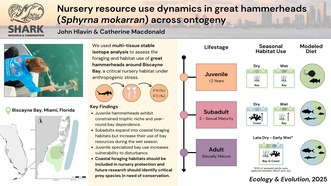

## 1 Introduction

Coastal sharks have seen major population declines over the past 50 years (Roff et al. 2018) with larger‐bodied and shallower‐water species assessed as at greatest risk of extinction due to their value and accessibility to coastal fisheries (Dulvy et al. 2014, 2021). Hammerhead sharks (family Sphyrnidae) are a particularly high‐risk group that is considered a priority for improved fisheries management (Dulvy et al. 2017, 2021). Some researchers have suggested that a high degree of specialization in hammerheads associated with their cephalofoil head morphology, their physiological adaptations for burst swimming, their aggregating and schooling behaviors, and their dependence on coastal ecosystems exacerbates their extinction risk and vulnerability to anthropogenic threats (Gallagher et al. 2014). The great hammerhead ( *Sphyrna mokarran* ), the largest hammerhead species, has been classified globally as Critically Endangered by the IUCN (Rigby et al. 2019), and early indicators of increasing regional population trends in the Western Atlantic population have only recently been reported (SEDAR 2023).

The conservation of threatened shark species like the great hammerhead necessitates not only effective fisheries management but also protection of essential habitats (Birkmanis et al. 2020; Heithaus 2007). Great hammerheads make long‐distance migrations and movements into pelagic waters (Guttridge et al. 2017) but generally prefer shallow coastal shelf habitats less than 30 m in depth, with core habitat‐use areas in the Western Atlantic that largely occur within Florida state waters (Casselberry et al. 2025; Graham et al. 2016; Guttridge et al. 2022). The importance of Florida's nearshore habitats to great hammerheads increases their exposure to anthropogenic threats like fishing, coastal development, and habitat loss. In the Florida Keys, great hammerheads have been shown to shift habitat use to increase spatiotemporal overlap with prey species targeted as prized game fish, elevating the risks associated with potential antagonistic encounters with recreational fishers (Casselberry et al. 2024; Griffin et al. 2022). Interactions with both recreational and commercial fisheries can lead to at‐vessel and post‐release mortality due to their heightened physiological stress response to capture (Binstock et al. 2023; Ellis et al. 2017; Gulak et al. 2015; Jerome et al. 2018).

**FIGURE 1:**
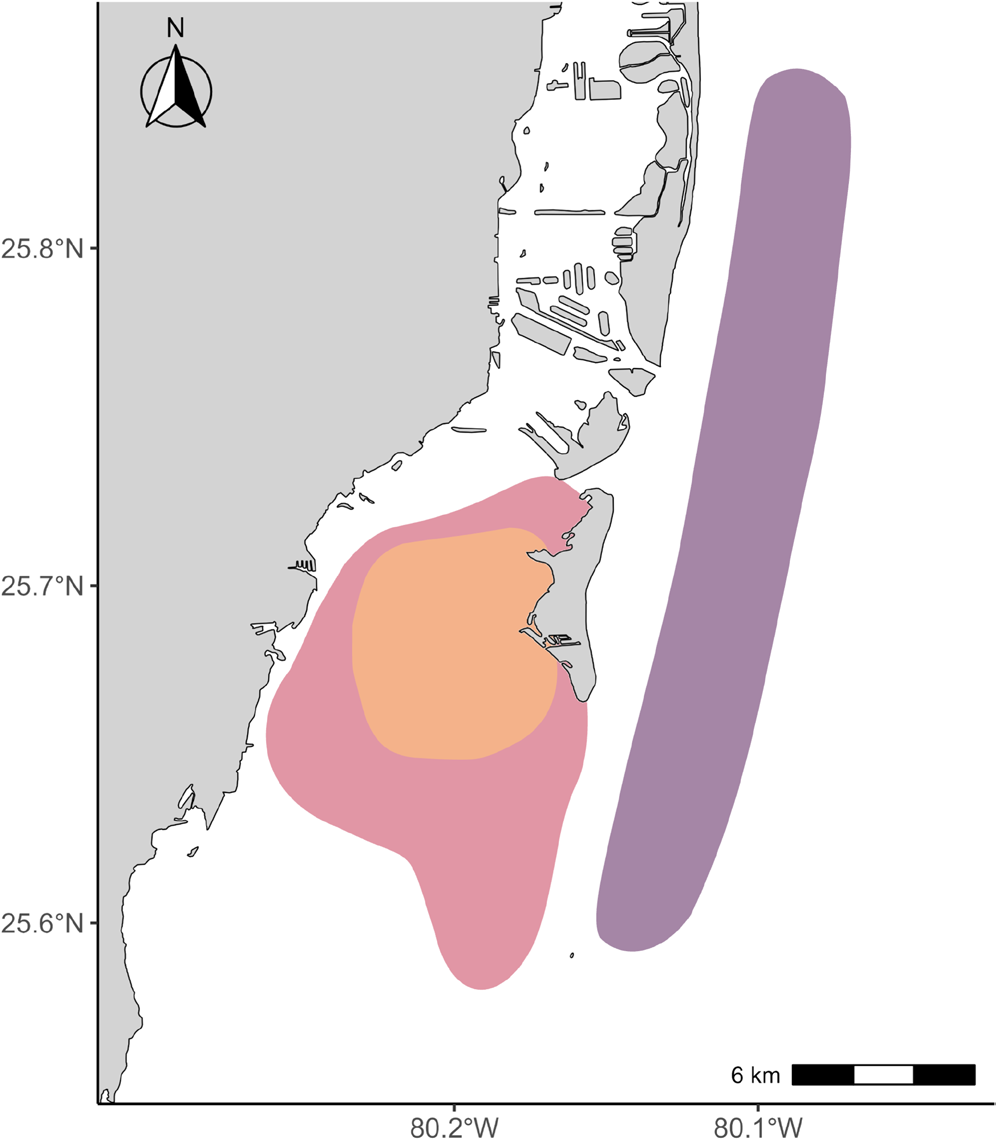
Locations of the sites in Biscayne Bay and nearby waters where great hammerheads were sampled. Orange represents the identified nursery site located off the southwest coast of Key Biscayne where most immature individuals were sampled, red represents transitional or non‐nursery bay habitats, and purple represents coastal habitats where most mature individuals were sampled. Map data from Florida Department of Transportation (2019).

Florida's shallow inshore and coastal waters are especially important to juvenile great hammerheads (Carlson et al. 2022; Macdonald et al. 2021). Biscayne Bay, Florida (Figure 1), in proximity to metropolitan Miami, is the first identified nursery area for great hammerheads on the east coast of the United States (Macdonald et al. 2021), although juveniles have been reported in other habitats (Barker et al. 2017; Carlson et al. 2022; Hueter and Tyminski 2007). Dependence on urbanized habitats in Biscayne Bay has previously been linked to poorer quality diets in juvenile sharks (De Sousa Rangel et al. 2021), which is likely exacerbated by the severe declines and degradation of Biscayne Bay's seagrass habitats (Carbonell et al. 2021; Santos et al. 2020). Therefore, closer investigation into the resource use patterns of great hammerheads associated with the Biscayne Bay nursery may prove valuable to our understanding of the constraints, trade‐offs, and threats impacting foraging during nursery association. This generates data needed for management efforts targeting the most essential habitats and prey resources of juvenile great hammerheads in South Florida to support the continued recovery of the regional population.

The use of nurseries in sharks is thought to enhance species fitness by promoting juvenile growth and survival to maturity through enhanced access to prey, reduced predation risk, or both (Heithaus 2007; Heupel et al. 2007; Heupel, Kanno, et al. 2019). There is growing evidence, however, that nursery dependence may require juveniles to accept trade‐offs in access to prey to reduce predation risk (Heithaus 2007; Heupel, Kanno, et al. 2019; Heupel, Munroe, et al. 2019; Matich and Heithaus 2015). Studies on the trophodynamics of shark nurseries have documented nursery‐associated juveniles having to contend with limited food availability and starvation risk (Bush and Holland 2002), constrained foraging opportunities to avoid predation risk (Bullock et al. 2024; Guttridge et al. 2012; Heupel and Hueter 2002), and interspecific competition for shared resources (Besnard et al. 2024; Kinney et al. 2011; Matich et al. 2017; Weideli et al. 2023).

Potentially as a strategy to overcome unreliable access to resources or unskilled foraging, some sharks become increasingly specialized as they grow, displaying broader generalist dietary niches and more opportunistic resource use as juveniles than as adults (Carlisle et al. 2015; Grubbs 2010; Heupel et al. 2014; Munroe et al. 2014; Newman et al. 2012). In other contexts, juveniles may exhibit smaller, relatively specialized dietary niches compared to older individuals, capitalizing on improved foraging efficiency on particular prey or reduced competition for it before expanding their dietary niche with ontogeny as new resources are exploited, often with minimal elimination of previously used resources (Afonso and Hazin 2015; Besnard et al. 2024; Cerutti‐Pereyra et al. 2022; Grubbs 2010; Munroe et al. 2014). Regardless of long‐term generalization or specialization, juvenile sharks may also manage resource constraints or instability through short‐term trophic plasticity—the ability to shift resource use over relatively short time periods or between different community contexts to take advantage of short‐term prey pulses or changes in habitat, predation risk, or competition (Lipscombe et al. 2024; Logan et al. 2020; Matich and Heithaus 2014; Matich et al. 2015, 2017). Therefore, juvenile resource use patterns across both ontogeny and season need to be considered in the identification and management of shark nurseries and key resources within them.

To date, the ontogenetic and seasonal resource use patterns of great hammerheads are poorly understood. Adult great hammerheads have been reported to be elasmobranch specialists that predominantly prey on rays, skates, and other sharks in addition to teleost prey (De Bruyn et al. 2021; Gallagher and Klimley 2018; Lubitz et al. 2023; Mourier et al. 2013; Raoult et al. 2019; Roemer et al. 2016); however, the ontogenetic timing of specialization on elasmobranch prey has not yet been extensively investigated. The only available information on juvenile diet comes from the Arabian Gulf, where stomach contents indicated juveniles are dietary specialists shifting from an exclusively teleost diet to substantial exploitation of batoid prey by their third year of life (Hsu et al. 2022). Recently, stable isotope analysis of Arabian Gulf juveniles concluded that they exhibit a strong dependence on nearshore shallow‐water foraging habitats and found no effect of sex or body size on trophic position (Lin et al. 2024). Ontogenetic shifts in resource use of great hammerheads in Australia have also been inferred from vertebral stable isotopes, which did indicate a general increase in trophic position with ontogeny (Raoult et al. 2019). Individuals also showed high levels of variation in the food webs within which they forage, ranging from coastal to pelagic to benthic, with little evidence of individuals switching between food webs across ontogeny (Raoult et al. 2019). In terms of seasonality, adult great hammerheads have been shown to exhibit seasonal residency in parts of Florida and The Bahamas (Guttridge et al. 2017) and a recent study reported that adults in the southeastern United States shift from using inshore habitats during spring and summer months to associations with offshore reefs from the summer through the winter (Casselberry et al. 2025).

Ontogenetic and seasonal resource use patterns have been comparatively well‐studied in the scalloped ( *S. lewini* ) and smooth hammerheads ( *S. zygaena* ; Besnard et al. 2023, 2024; Cerutti‐Pereyra et al. 2022; Coiraton et al. 2020; Estupiñán‐Montaño, Galván‐Magaña, et al. 2021; Estupiñán‐Montaño, Tamburin, et al. 2021; Hoyos‐Padilla et al. 2014; Logan et al. 2020; Rosende‐Pereiro et al. 2020; Torres‐Rojas et al. 2014, 2015). Juvenile scalloped hammerheads have been reported in multiple studies to be generalist and opportunistic predators (Rosende‐Pereiro et al. 2020; Torres‐Rojas et al. 2014, 2015) before undergoing a niche contraction with ontogeny hypothesized to suggest reproductive‐age females specializing in nutritious and easily captured coastal prey like mangrove fish and crustaceans (Estupiñán‐Montaño, Galván‐Magaña, et al. 2021; Estupiñán‐Montaño, Tamburin, et al. 2021). Elsewhere, juvenile scalloped and smooth hammerheads sharing habitats exhibit smaller, more specialized niches—potentially a response to reduce competition between these ecologically similar species by partitioning resources (Besnard et al. 2024)—and may then expand niches as they grow (e.g., Cerutti‐Pereyra et al. 2022). Seasonal shifts in habitat use have also been documented in juvenile smooth hammerheads, which migrate between restricted summer and winter core areas, a potential strategy to improve foraging opportunities associated with warmer sea surface temperatures and higher primary productivity (Logan et al. 2020). Given the relative paucity of resource use information for great hammerheads, especially juveniles in the Western Atlantic, further investigation into ontogenetic and seasonal resource use patterns is vital to improving our understanding of the trophic ecology of great hammerheads, how it may differ from their relatives, and the extent of juvenile dependence on an inshore nursery habitat.

This study characterizes resource use patterns of great hammerheads around the Biscayne Bay nursery to investigate diet and foraging habitat across ontogeny and season using stable isotope analysis (SIA) of muscle and plasma samples. SIA is a well‐established and typically minimally invasive methodology to gain insight into a shark's feeding and movement ecology, making it a useful tool for conservation of threatened elasmobranch species (reviewed in Shiffman et al. 2012). Isotopes in marine food webs behave predictably such that the ^13^C/^12^C and ^15^N/^14^N ratios (expressed as *δ* ^13^C and *δ* ^15^N values in ‰) can serve as proxies for foraging habitat and trophic position (Hussey, MacNeil, et al. 2012). Specifically, baseline *δ* ^13^C values vary substantially across South Florida's primary producers (reviewed in Shiffman et al. 2019) with estuarine and shallow‐water seagrass beds having a higher *δ* ^13^C value relative to the algae and corals found on coastal reefs (Behringer and Butler 2006; Kieckbusch et al. 2004; Lamb et al. 2012; Swart et al. 2005), and all coastal environments higher than the pelagic phytoplankton of the North Atlantic (Mompeán et al. 2013). This spatial variation in baseline *δ* ^13^C values and minimal fractionation from diet to consumer allows *δ* ^13^C values to be an indicator of estuarine, coastal, or pelagic foraging by consumers (Hussey, MacNeil, et al. 2012). Baseline *δ* ^15^N values, however, do not vary substantially among local primary producers (Behringer and Butler 2006; Kieckbusch et al. 2004; Lamb et al. 2012; Mompeán et al. 2013) but fractionate more substantially from diet to consumer, such that *δ* ^15^N values increase iteratively with rising trophic level (Hussey, MacNeil, et al. 2012).

Additionally, by analyzing multiple tissues in tandem, researchers can choose to integrate and/or compare short‐term and longer‐term resource use in analyses based on varying isotopic incorporation rates between tissue types (Li et al. 2024; Matich and Heithaus 2014; Matich et al. 2015; Reum and Essington 2013). Specifically, plasma has been shown to have a rapid isotopic incorporation rate—estimates range from as fast as ~7 days in juvenile sharks (Tamburin et al. 2024) to ~1 month (Kim, Del Rio, et al. 2012)—meaning plasma provides insight into near real‐time diet. Muscle, however, incorporates isotopes much more slowly across several months, offering a more stable time‐averaged picture of resource use (Hussey, MacNeil, et al. 2012; Kim, Del Rio, et al. 2012; Li et al. 2024; Matich et al. 2015).

Using multi‐tissue SIA, this study sought to answer two questions about the trophic ecology of great hammerheads in South Florida: First, do great hammerheads exhibit an ontogenetic shift in resource use consistent with dependence on the Biscayne Bay nursery as juveniles? And second, do great hammerheads shift foraging habitat use seasonally? Answers will contribute to our knowledge of how this Critically Endangered species uses resources within an essential but highly anthropogenically threatened habitat.

## 2 Materials and Methods

### 2.1 Study Location

Sampling took place year‐round from 2018 to 2025 in Biscayne Bay, Florida (25.7° N, 80.2° W) and nearby coastal waters around the identified nursery site of great hammerheads (Macdonald et al. 2021; Figure 1). Biscayne Bay is a marine estuary adjacent to the urbanized coast of metropolitan Miami with habitats including mangroves, seagrass beds, mud flats, and patch reefs (Roessler and Beardsley 1974). The habitat continuum is completed outside estuary boundaries with the forereefs of the Florida Reef Tract in the coastal waters of the Western Atlantic (Lirman et al. 2003). Biscayne Bay and the adjacent habitats host a biodiverse faunal community including more than 30 endangered species or species of concern (Cantillo et al. 2000) and over 100 species of importance to local recreational and commercial fisheries (Stoa 2016). Unfortunately, these habitats have seen significant alterations and habitat declines from anthropogenic impacts of urbanization, overfishing, physiochemical changes, and destruction to and die‐offs of seagrasses and corals (Cantillo et al. 2000; Carbonell et al. 2021; Santos et al. 2020; Toth et al. 2022).

### 2.2 Sample Collection

Capture and sampling methodologies followed those described in Macdonald et al. (2021). Great hammerhead sharks were captured during ongoing shark survey efforts via modified scientific bottom longline (max of two lines in use simultaneously, 130 m/22 hooks per line) and weighted marked drumlines (max of ten in use simultaneously) using baited non‐stainless steel circle hooks (13/0–16/0 size). All fishing gear was deployed with a target soak time of 60 min. Scientific workups took place on a partially submerged platform for large sharks or aboard the deck of the research vessel for small sharks. Upon landing, sharks immediately received ambient seawater for respiration using a custom PVC ventilation system and were frequently bucketed throughout the workup to keep skin and gills wet and minimize the risk of dehydration, overheating, or air‐related gill injury.

During workups, we measured three standard lengths: pre‐caudal length (PCL; snout to pre‐caudal pit), fork length (FL; snout to tail fork) and stretched total length (TL; snout to tip of the tail). We also measured frontal span (i.e., “girth”: rear of right pectoral fin to rear of left pectoral fin) and cephalofoil (head) width. Each animal received a unique external mark‐recapture identification tag. Biological samples for this study consisted of a ~4 mm biopsy of white muscle from the dorsal flank using a sterile biopsy punch and < 8 mL of blood drawn via caudal venipuncture and stored in lithium heparin‐coated tubes (shown to have no effect on SIA; Kim and Koch 2012) to prevent clotting. At the end of each sampling day, we centrifuged whole blood to separate plasma from other blood components and stored all samples in a −20°C freezer until SIA, as recommended by Kim and Koch (2012).

Other elasmobranch species encountered during ongoing shark survey efforts, as well as teleost bycatch, were similarly worked up, sampled, and analyzed for future trophic ecology investigations. All research activities were conducted under the following institutional approvals and sampling permits: University of Miami IACUC protocol #20‐043, #23‐173, Florida Fish and Wildlife Commission #SAL‐23‐1798A‐SRP, and National Park Service #BISC‐2023‐SCI‐0029.

### 2.3 Stable Isotope Analysis

Muscle biopsies were rinsed with e‐pure water, separated from any skin or connective tissue, and dried at 40°C for ~48 h. Aliquots of ~0.3–0.5 mL of plasma were dried at 40°C in a weigh boat for 2–4 h, scraped free, and dried further for ~48 h. All dried samples were manually homogenized into a powder via mortar and pestle. We performed lipid‐ and urea‐extraction on all elasmobranch samples, a methodological best practice recommended by Kim and Koch (2012) to account for variable amounts of lipids and urea across elasmobranch species and tissue types, which can bias *δ* ^13^C and *δ* ^15^N values (Bennett‐Williams et al. 2022; Carlisle et al. 2017; Crook et al. 2019; Hussey, Olin, et al. 2012; Kim and Koch 2012). No extractions were performed on teleost samples and no further mathematical corrections were deemed necessary (Peterson et al. 2020; Post et al. 2007).

For muscle lipid‐ and urea‐extractions, we employed the methods described in Kim and Koch (2012). Lipid extraction of dried muscle consisted of two rinses in 10 mL of petroleum ether followed by urea extraction comprising three rinses in 10 mL of e‐pure water, with each of the five total rinses involving a 15‐min sonication period. After sonication, the rinse solvent was gently decanted before the next solvent was added. After the final water rinse, the extracted muscle samples were once again dried at 40°C for ~48 h.

For plasma, a different extraction method was used to minimize potential bias due to the risk of losing plasma's free amino acids attributed to water rinses (Kim and Koch 2012). Following the methods of Martins et al. (2022), we performed extraction for plasma using a 2:1 chloroform:methanol solvent capable of extracting both lipids and urea (Hussey, Olin, et al. 2012), removing the need for a water rinse. Plasma samples were immersed in 5 mL of the solvent, agitated for 30 s, and left in a 30°C water bath for 24 h, after which the solvent was decanted, and the steps repeated a second time. After extraction, plasma samples were left in a fume hood for ~24 h to allow any remaining solvent to evaporate. ~0.3–0.5 mg of dried tissue material was weighed out and packed into tin capsules for analysis.

Carbon and nitrogen stable isotope ratios were analyzed using a Costech elemental combustion system interfaced to a Thermo Delta V Advantage isotope ratio mass spectrometer in the Stable Isotope Lab of the University of Miami Rosenstiel School and recorded in *δ* notation according to Equation (1)

(1) $$\delta^{h}X=\left(\frac{R_{\text{sample}}}{R_{\text{standard}}}-1\right)\times 1000$$

where R is the ratio of the minor isotope to the major isotope (i.e., ^13^C/^12^C and ^15^N/^14^N) and Vienna Pee Dee Belemnite and atmospheric nitrogen are the standards for carbon and nitrogen, respectively. Each run consisted of two procedural blanks bookending 35 samples interspersed with 13 internal standards (glycine and acetanilide) for quality control. Samples were corrected to glycine, and external precision was calculated as the mean of the per‐run standard deviations of glycine: ± 0.12‰ (range: 0.03‰–0.22‰) for *δ* ^15^N and ± 0.07‰ (0.01‰–0.15‰) for *δ* ^13^C.

### 2.4 Data Analyses

All statistical tests and models were run in RStudio (R 4.4.2; R Core Team 2024), the code for which is publicly available, using an *α*‐significance level of 0.05. Sizes‐at‐maturity of 187 cm FL for males and 224 cm FL for females were used to assign maturity to our sampled individuals as reported for the north‐western Atlantic Ocean and Gulf of Mexico region (Piercy et al. 2010). The wet season was analyzed as May 1st to October 31st and the dry season as November 1st to April 30th. The two smallest hammerheads encountered at the nursery site (63 and 67 cm FL) had lower *δ* ^13^C values in both tissues relative to the other small nursery‐site hammerheads (see Section 3), potentially indicating continued reliance on maternally provisioned energy stores (Hussey, Wintner, et al. 2010). We elected to omit these two individuals from the rest of the statistical analyses owing to the risk of maternal bias.

We first tested for differences in mean *δ* ^13^C and *δ* ^15^N values for both muscle and plasma by sex (male vs. female) and maturity (immature vs. mature), using ANOVAs when assumptions of normality and homoscedasticity were upheld (Shapiro–Wilk and Levene tests, respectively) or Wilcoxon rank‐sum tests when assumptions were violated. Season (wet vs. dry) was also included as a factor in the ANOVAs for plasma only, given its rapid turnover period that allows for detection of short‐term dietary changes, and all ANOVAs were fully crossed to test for interaction effects between sex and maturity (and season, for plasma only). We then performed ordinary least squares linear regression to evaluate the relationships between fork length and *δ* ^13^C and *δ* ^15^N values for each tissue type.

To more closely investigate ontogenetic and seasonal resource use patterns, we constructed a Bayesian stable isotope mixing model as the foundation of the remaining analyses using the ‘MixSIAR’ package (Stock et al. 2018) to estimate the relative contributions of four potential prey sources. Each model endmember was composed of a single representative taxon sampled from the coastal or inshore habitats of South Florida: bay batoids (southern stingray *Hypanus americanus*; *n* = 8, *δ* ^15^N = 11.6‰ ± 1.2‰, *δ* ^13^C = −13.2‰ ± 1.0‰), bay teleosts (gray snapper *Lutjanus griseus* ; *n* = 4, *δ* ^15^N = 8.7‰ ± 0.5‰, *δ* ^13^C = −13.5‰ ± 1.4‰), coastal sharks (Atlantic sharpnose shark *Rhizoprionodon terraenovae* ; *n* = 24, *δ* ^15^N = 12.5‰ ± 0.5‰, *δ* ^13^C = −15.2‰ ± 0.8‰), and coastal teleosts (almaco jack *Seriola rivoliana* ; *n* = 8, *δ* ^15^N = 9.8‰ ± 1.6‰, *δ* ^13^C = −16.3‰ ± 0.8‰). Elasmobranch endmember species were sampled and analyzed during the present study, while teleost endmember data were derived primarily from the literature (Blanar et al. 2021; Vaslet et al. 2012) as non‐elasmobranch bycatch was minimal due to gear selectivity. A pairwise PERMANOVA with Bonferroni‐adjusted *p*‐values and a PERMDISP (*n* = 9999 permutations for both tests) were performed using the ‘pairwiseAdonis’ (Martinez Arbizu 2017) and ‘vegan’ (Oksanen et al. 2024) packages to assess whether isotopic differences among endmembers reflected true location differences not confounded by differences in dispersion.

Mixing models require an estimation of the isotopic fractionation between an organism's diet and its tissue, or the diet‐tissue discrimination factor (DTDF). DTDFs have been shown to vary between tissues and can be species‐ and diet‐dependent (Caut et al. 2013; Kim, Del Rio, et al. 2012). Species‐specific DTDFs are often unavailable for the species and/or its tissue types analyzed in a study, and so, despite taxonomic dissimilarities, many studies (e.g., Li et al. 2024) opt to apply published DTDF estimates from long‐term controlled feeding studies on captive sharks (e.g., Caut et al. 2013; Hussey, Brush, et al. 2010; Kim, Casper, et al. 2012; Kim, Del Rio, et al. 2012; Malpica‐Cruz et al. 2012). To the best of our knowledge to date, Kim, Casper, et al. (2012) and Kim, Del Rio, et al. (2012) are the only studies that estimate the DTDF of an elasmobranch's muscle and plasma simultaneously, using captive leopard sharks ( *Triakis semifasciata* ). Their studies, however, did not perform urea and lipid extractions on the plasma, which can have significant isotopic effects on elasmobranch plasma (Bennett‐Williams et al. 2022; Crook et al. 2019). Considering the difference in sample preparation and the substantial ecological dissimilarities between great hammerheads and leopard sharks, we opted to universally apply a DTDF published for shark muscle by Hussey, Brush, et al. (2010) using more ecologically similar large‐bodied apex predatory shark species, following Raoult et al. (2019). To apply this muscle DTDF to plasma, however, we first corrected plasma values to the muscle values using the average difference between paired samples, such that plasma *δ* ^13^C values were corrected by −0.70‰ and *δ* ^15^N values by +2.22‰. These inter‐tissue correction factors are more comparable to the average isotopic offset between extracted muscle and extracted plasma of other elasmobranchs (see Crook et al. 2019) than they are to the difference between muscle and plasma DTDFs (−1.1‰ for *δ* ^13^C and 1.5‰ for *δ* ^15^N) published by Kim, Casper, et al. (2012). To confirm that these inter‐tissue correction factors do not change across the range of *δ* ^13^C and *δ* ^15^N values observed, we fit linear models of paired muscle and plasma values for each isotope per season (given the sensitivity of rapid‐turnover plasma to seasonal diet shifts) and conducted *t*‐tests to evaluate if the slopes differed significantly from 1. No significant difference was interpreted to suggest a 1:1 relationship that does not vary substantially with shark diet, supporting the validity of the mean difference correction (see Matich and Heithaus 2014). Muscle and corrected plasma values were then provided to the mixing model with the DTDF set at 0.9‰ ± 0.33‰ for *δ* ^13^C values and 2.29‰ ± 0.22‰ for *δ* ^15^N values per Hussey, Brush, et al. (2010).

We simulated 95% density clouds for each endmember using 1000 random draws from independent normal distributions based on the mean and standard deviation of *δ* ^13^C and *δ* ^15^N to confirm all DTDF‐corrected great hammerhead values fell within the isotopic mixing space. Individual‐level diets were then modeled in 44 great hammerheads with paired muscle and plasma samples, such that the *n* = 2 per individual allowed for individual to be modeled as a random effect. Modeling short‐turnover plasma and long‐turnover muscle together was therefore assumed to provide a time‐integrated picture of resource use for each modeled individual. The model was supplied with a Dirichlet generalist prior with the alpha parameter set to 1 (i.e., a prior that assumes proportional contributions from each source that sum to 100%). The mixing model simulated samples to generate posterior distributions using a Markov chain Monte Carlo (MCMC) algorithm consisting of 3 chains 300,000 iterations in length with a burn‐in period of 200,000 and a thinning rate of 100, resulting in 3000 total posterior samples for each source per individual. Convergence was assessed using Gelman‐Rubin diagnostics (R‐hat), and the precision of estimates was evaluated using the Geweke diagnostic. Dietary contributions were estimated as the posterior means for each source at the individual level, preserving the compositional constraints of the Dirichlet distribution. Bay and coastal source estimates were also combined a posteriori (Stock et al. 2018) to evaluate habitat‐specific resource use.

#### 2.4.1 Ontogenetic Resource Use

To investigate ontogenetic shifts in resource use, we evaluated changes in diet contribution across fork length (FL) by both prey group and habitat. We used the ‘brms’ package (Bürkner 2017) to fit Bayesian Dirichlet regression models, with FL included as a smooth predictor of dietary contributions and the Dirichlet likelihood constraining the response such that all dietary contributions sum to one at every point along the FL spectrum. Each model consisted of 4 chains 4000 iterations in length, with a 2000 iteration warm‐up. Convergence of the model coefficients was evaluated by inspecting R‐hat and estimated sample size (ESS) values (Vehtari et al. 2021).

We also performed breakpoint analysis using the ‘strucchange’ package (Zeileis et al. 2002) to detect significant changes in the mean, where the optimal number and location of breakpoints was determined using lowest Bayesian Information Criterion (BIC) with a minimum segment length of 5 data points. Based on the results of the habitat resource use breakpoint analysis, we classified immature individuals (determined using published sex‐specific sizes‐at‐maturity in Piercy et al. 2010) into two emergent age classes: juveniles—pre‐breakpoint immature individuals with high bay (i.e., nursery) resource use; and subadults—post‐breakpoint immature individuals exhibiting mixed bay and coastal resource use (see Section 3). This generated three total age classes for analysis: breakpoint‐determined juvenile and subadult groups, and an adult group composed of mature individuals.

Finally, we used the ‘SIBER’ package (Jackson et al. 2011) to estimate the size of the area of isotopic space occupied by each age class (hereafter “isotopic niche”) as a proxy for ecological niche. Ontogenetic changes in isotopic niche were assessed by quantifying the Bayesian standard ellipse area (SEAb; representing the mean of 4000 posterior samples) from each age class's muscle values (due to their insensitivity to short‐term fluctuations) and uncertainty was calculated as the 95% Highest Density Region (HDR) using the ‘hdrcde’ package (Hyndman et al. 2021). Bayesian probabilities (i.e., Bayesian *p*‐values) were calculated from pairwise comparisons of each age class's posterior samples to test for significant differences in SEAb estimates according to Equation (2)

(2) $$p\left({\text{SEAb}}_{1}>{\text{SEAb}}_{2}\;\right)=\frac{\sum_{i=1}^{N}I\left({\text{SEAb}}_{1,i}>{\text{SEAb}}_{2,i}\;\right)}{N}$$

where *p* is the probability that SEAb_*1*_ is greater than SEAb_2_, *N* is the total number of posterior samples (i.e., 4000), *I*(·) is an indicator function that equals 1 when true and 0 otherwise, and _1,*i*_ and _2,*i*_ are the *i*th posterior samples for SEAb_1_ and SEAb_2_, respectively (Jackson et al. 2011). We then quantified similarities between the isotopic niches of each size class by calculating the mean overlap between posterior SEAb samples (Jackson et al. 2011).

#### 2.4.2 Seasonal Resource Use

To investigate seasonal shifts in resource use, we again fit Bayesian Dirichlet regressions of the dietary contributions by habitat with day of the year (DOY) included as a smooth predictor and conducted breakpoint analysis, as described above. To isolate the effect of season from the effect of ontogeny, analyses were performed on each age class independently. Since habitat contributions were modeled from both muscle (long‐term) and plasma (short‐term) isotope values, to confirm if observed seasonal differences in resource use represented true shifts in foraging habitat rather than differences in specialist trophic strategies between individuals, we fit generalized additive models (GAMs) of the difference between plasma and muscle *δ* ^13^C values (hereafter, “inter‐tissue difference”) with DOY as a smooth term for each age class using the ‘mgcv’ package (Wood 2017). For the juvenile GAM, DOY values covered nearly the entire 365‐day periodicity of a year, allowing the model to be fit with a cyclic spline after the addition of a pseudo‐datapoint for day 365 weighted at 10^−10^ relative to weights of 1 for the data. Due to the difference in turnover rates between the two tissues (~1 month or less in plasma vs. ~6 months in muscle; Kim, Del Rio, et al. 2012; Tamburin et al. 2024), comparison of dynamic plasma *δ* ^13^C values to stable muscle *δ* ^13^C values can be used to assess short‐term trophic changes and the direction and magnitude of seasonal shifts in foraging habitat (Matich and Heithaus 2014; Matich et al. 2015). Therefore, inter‐tissue differences greater or less than the expected difference of 0.70‰ (mean inter‐tissue difference between all paired samples) were considered to suggest a short‐term increase or decrease in use of higher‐*δ* ^13^C inshore foraging habitats, respectively. The larger the magnitude of the inter‐tissue difference relative to 0.70‰, the more pronounced the foraging shift was assumed to be, while differences approximately equal to 0.70‰ were considered to indicate short‐term foraging relatively consistent with long‐term foraging. Finally, as described above, we also used the plasma values to calculate and compare dry versus wet season SEAb for each age class and quantified the overlap between them to investigate seasonal differences in isotopic niche as percent of the total area occupied in both seasons combined: overlap/(SEAb_dry_ + SEAb_wet_–overlap).

## 3 Results

A total of 62 biopsies, sampled from 2018 to 2025, were analyzed from great hammerhead sharks ranging in size from 63 to 287 cm FL captured in Biscayne Bay and nearby coastal waters (Figure 1), 46 of which also provided paired plasma samples (63–264 cm FL; Appendix 1). Mature individuals were captured and sampled primarily in coastal habitats, three were sampled from transitional or non‐nursery bay habitats, and none at the identified nursery site, while 23 of 34 immature individuals were captured and sampled at the identified nursery site (Figure 1).

**FIGURE 2:**
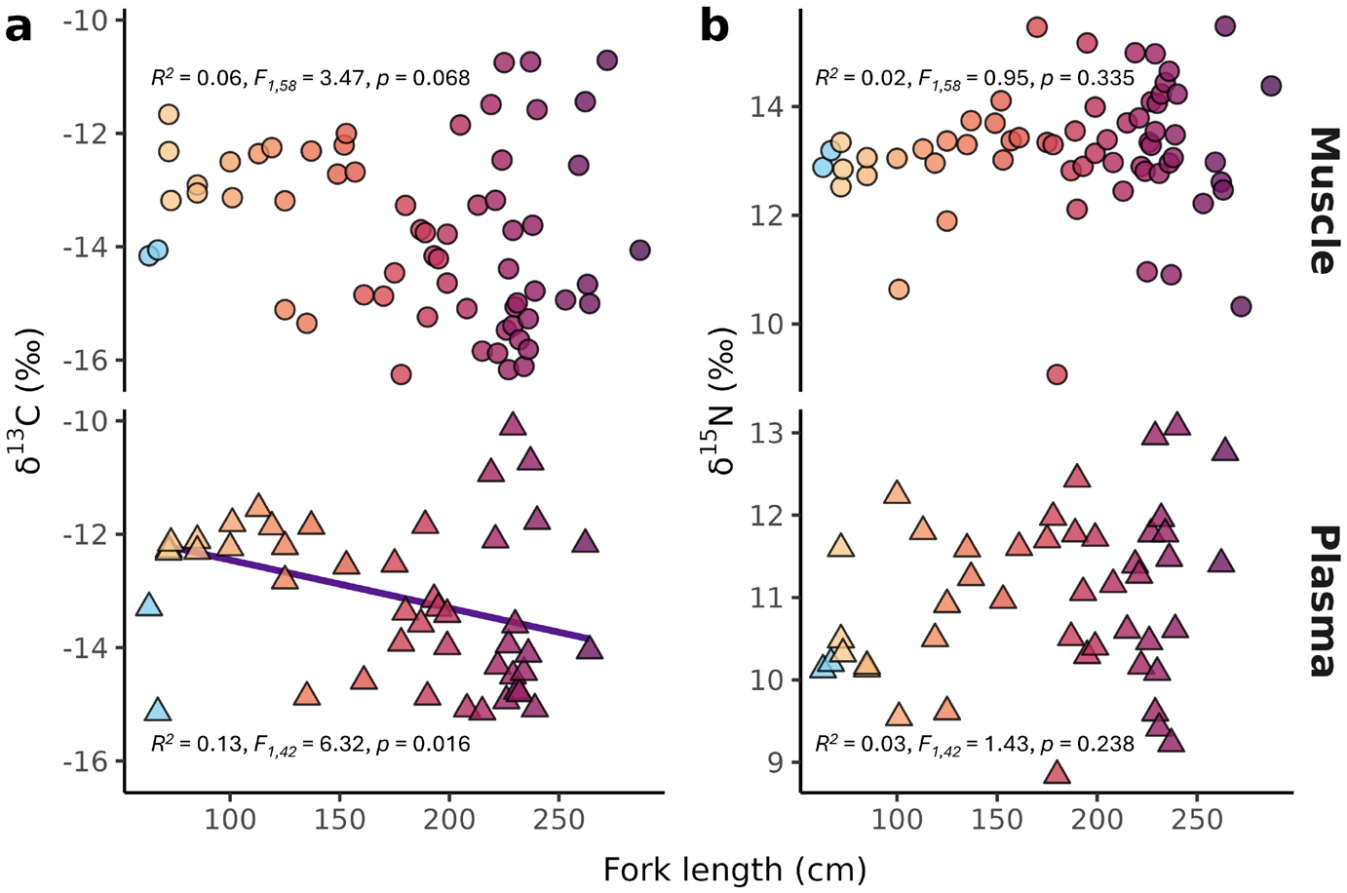
Great hammerhead (a) *δ* ^13^C and (b) *δ* ^15^N values of muscle (circles; top) and plasma (triangles; bottom) versus FL. Light‐to‐dark color gradient represents increasing size in FL, except for two light blue points corresponding to the two smallest sampled individuals omitted from statistical analyses due to suspected lingering maternal influence. Results of ordinary least squares linear regressions are annotated, and linear fit was depicted when significant.

A full summary of the isotope ratios broken down by demographic and season is available in Appendix 1. Mean C:N ratios of muscle and plasma were 3.35 (± 0.09) and 3.61 (± 0.09), respectively, consistent with our expectations for lipid‐ and urea‐extracted muscle and plasma protein (Crook et al. 2019). Muscle *δ* ^15^N values did not differ significantly by sex (*W* = 453, *p* = 0.971) or maturity (*W* = 469, *p* = 0.927), based on Wilcoxon rank‐sum tests (assumption of normally distributed ANOVA residuals was not met: Shapiro–Wilk, *W* = 0.93, *p* = 0.003). Plasma *δ* ^15^N values did not differ significantly by sex (*F*_1,36_ = 0.69, *p* = 0.412), maturity (*F*_1,36_ = 0.27, *p* = 0.605), or season (*F*_1,36_ = 0.86, *p* = 0.359). Muscle *δ* ^13^C values did not differ by sex (*F*_1,56_ = 0.39, *p* = 0.534) or maturity (*F*_1,56_ = 1.39, *p* = 0.243). Plasma *δ* ^13^C values did not differ significantly by sex (*F*_1,36_ = 0.11, *p* = 0.742), maturity (*F*_1,36_ = 2.73, *p* = 0.107), or season (*F*_1,36_ = 4.01, *p* = 0.053). No interaction terms between sex, maturity, and/or season were significant in any model (*p* > 0.05). Ordinary least squares linear regression indicated that there were no significant relationships between fork length and the *δ* ^13^C values of muscle (*R* ^*2*^ = 0.06, *F*_1,58_ = 3.47, *p* = 0.068) or the *δ* ^15^N values of either tissue (muscle: *R* ^2^ = 0.02, *F*_1,58_ = 0.95, *p* = 0.335; plasma: *R* ^*2*^ = 0.03, *F*_1,42_ = 1.43, *p* = 0.238; Figure 2). There was, however, a significant but weak negative linear relationship with plasma *δ* ^13^C values (*R* ^2^ = 0.13, *F*_1,42_ = 6.32, *p* = 0.016; Figure 2a).

On average, muscle *δ* ^13^C values were 0.70‰ ± 0.87‰ lower than those of plasma, while muscle *δ* ^15^N values were 2.22‰ ± 0.95‰ higher than plasma. Muscle and plasma were significantly correlated for both isotopes in both the wet and dry seasons (*δ* ^15^N_dry_: *R* ^2^ = 0.29, *F*_1,20_ = 8.12, *p* = 0.010; *δ* ^15^N_wet_: *R* ^2^ = 0.42, *F*_1,20_ = 14.49, *p* = 0.001; *δ* ^13^C_dry_: *R* ^2^ = 0.85, *F*_1,20_ = 110.90, *p* < 0.001; *δ* ^13^C_wet_: *R* ^2^ = 0.55, *F*_1,20_ = 24.44, *p* < 0.001; Appendix 2). The slopes of these correlations were determined to not significantly differ from 1 via *t*‐tests (*δ* ^15^N_dry_: slope = 0.64, *t*_20_ = −1.60, *p* = 0.125; *δ* ^15^N_wet_: slope = 0.70, *t*_20_ = −1.65, *p* = 0.115; *δ* ^13^C_dry_: slope = 1.03, *t*_20_ = 0.31, *p* = 0.758; *δ* ^13^C_wet_: slope = 0.836, *t*_20_ = −0.97, *p* = 0.34), supporting the assumption that the mean difference between the tissues is constant across the spectrum of isotope values observed and seasons and validating our correction of plasma values to muscle values using the mean difference prior to applying the muscle DTDF in the mixing model.

**FIGURE 3:**
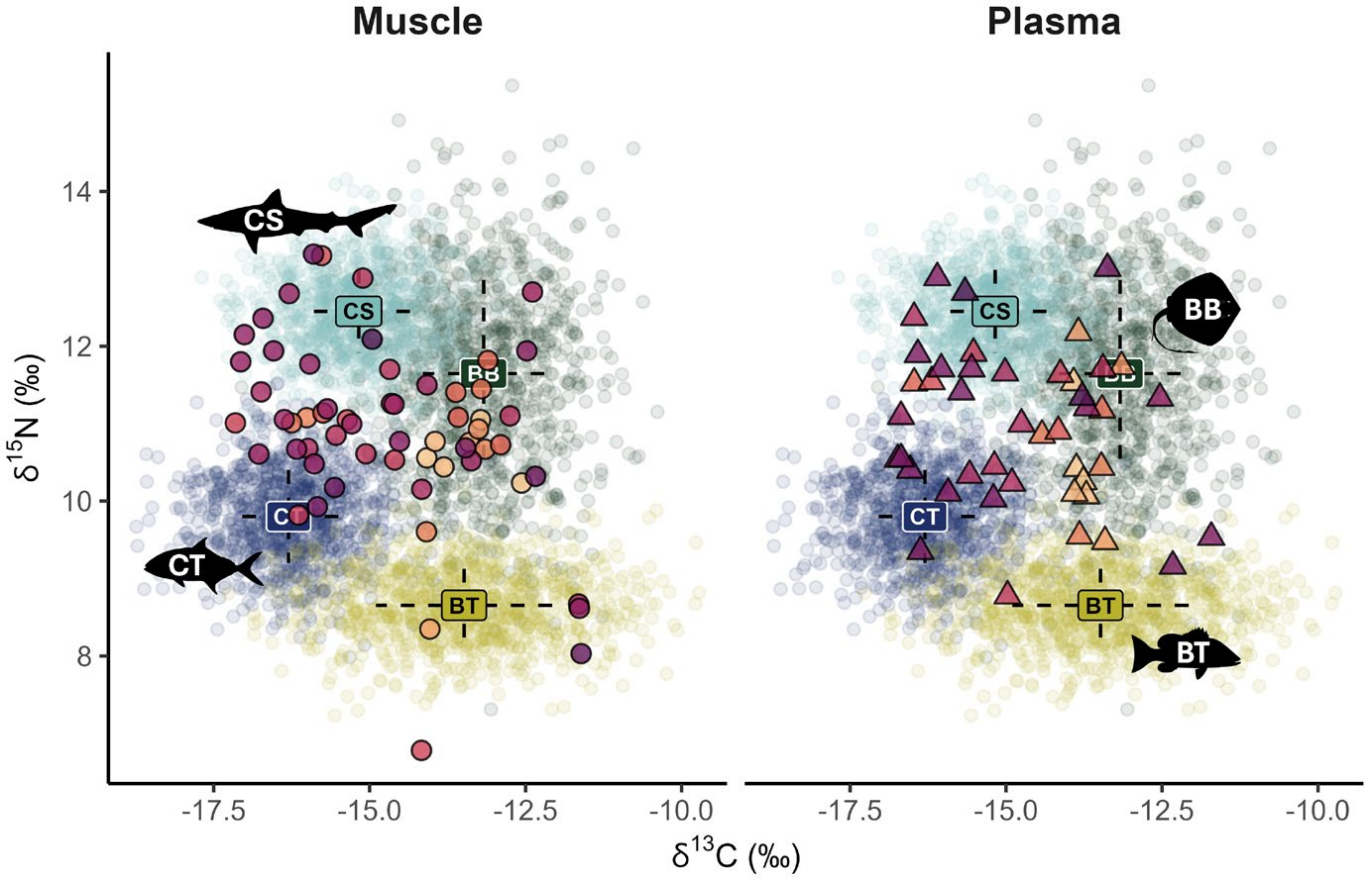
Isotope values from individuals with paired muscle and plasma samples included in mixing model (*n* = 44), plotted by tissue type where circles are muscle (left) and triangles are plasma (right) and light‐to‐dark gradient represents increasing FL. Weighted mean (± SD) and 95% density cloud of each prey source represent the isotope mixing space: bay batoids (BB: dark green), bay teleosts (BT: light green), coastal sharks (CS: light blue), and coastal teleosts (CT: dark blue).

All four mixing model endmembers were confirmed to occupy significantly different locations in isotopic space not due to differences in dispersion (PERMDISP: *F*_3,40_ = 1.44, *p* = 0.244; all pairwise PERMANOVA Bonferroni‐adjusted *p* < 0.013) and all DTDF‐corrected great hammerhead samples fell within the uncertainty of the isotopic mixing space (Figure 3). Gelman‐Rubin diagnostics (R‐hat values) of < 1.1 for all and < 1.05 for 403 of 404 estimated parameters confirmed acceptable mixing model convergence. For the three chains, Geweke diagnostics indicated that 6%, 5%, and 5% of parameters fell outside the ± 1.96 threshold (5% expected under convergence), suggesting only a minor deviation from ideal convergence in one chain consistent with chance or issues with the precision of a few parameters.

### 3.1 Ontogenetic Resource Use

**FIGURE 4:**
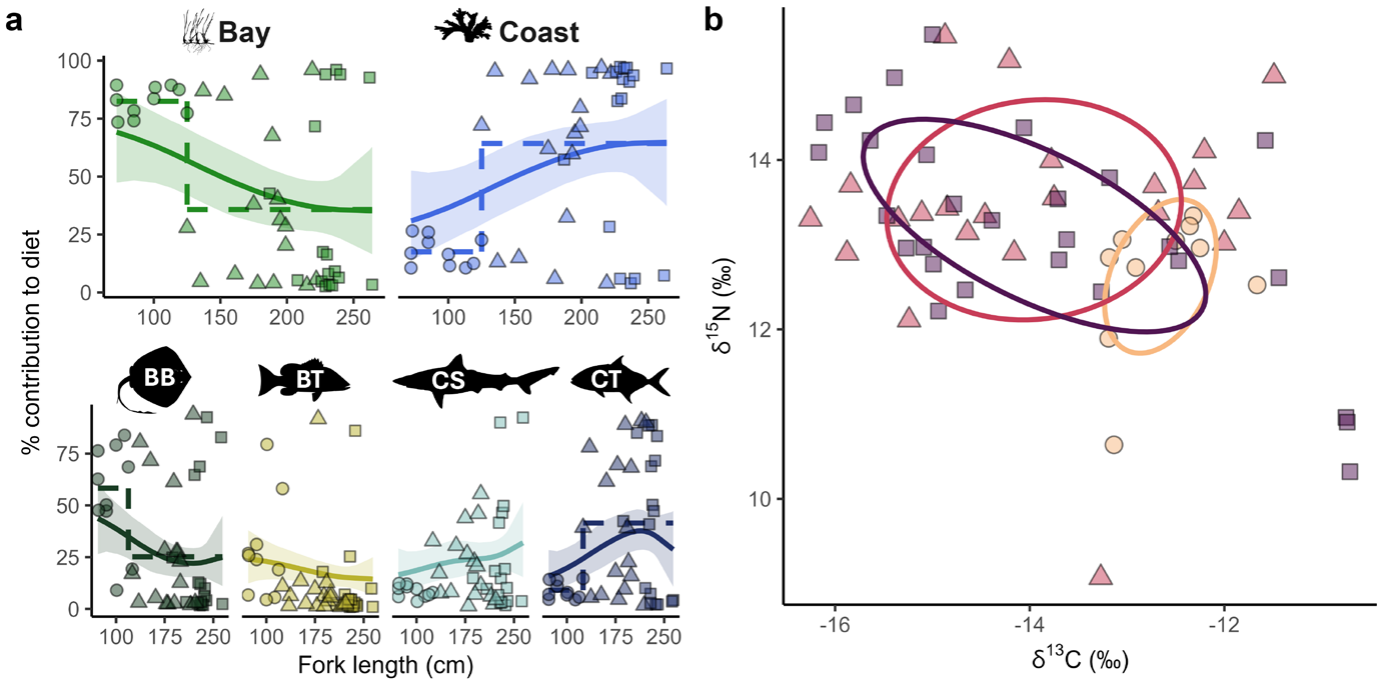
(a) Post hoc models of individual dietary contributions (points) predicted by the mixing model, with habitat (top) and prey source (bottom) contributions depicted by FL where BB, bay batoids; BT, bay teleosts; CS, coastal sharks; CT, coastal teleosts. Solid lines correspond to fitted Bayesian Dirichlet regressions, with 95% credible interval ribbon. Dashed lines represent significant breakpoints and the means before and after. Shapes denote emergent age classification based on the identified habitat use breakpoint—circles: juveniles (pre‐breakpoint immature); triangles: subadults (post‐breakpoint immature); and squares: adults (mature). (b) Standard ellipse areas from muscle values by age class, colored light‐to‐dark and with the same shape assignments as (a).

Dirichlet regressions of dietary contributions against FL by habitat and by diet group successfully converged according to R‐hat values of 1 and ESS values > 400 (Vehtari et al. 2021). Fitted smooth curves and their 95% credible intervals are displayed in Figure 4a. Breakpoint analysis detected one significant breakpoint according to BIC in the dietary contributions by habitat corresponding to 125 cm FL, as well as in the bay batoid and coastal teleost contributions corresponding to 119 and 125 cm FL, respectively (Figure 4a). Overall, these analyses indicate a decrease in bay contribution from ~70%–80% to ~30%–40% of diet with increasing FL largely due to decreased batoid contribution, with a notable shift to more coastal resource use around 125 cm largely due to increased coastal teleost contribution (Figure 4a). Using the breakpoint detected in habitat contributions, pre‐breakpoint immature individuals with high bay (i.e., nursery) resource use were classified as juveniles while post‐breakpoint immature individuals exhibiting a mix of bay and coastal resource use were classified as subadults.

Bayesian standard ellipse areas (SEAb) from muscle isotope values were 1.35 [95% HDR: 0.51–2.25]‰^2^ for juveniles, 5.87 [3.43–8.37]‰^2^ for subadults, and 5.09 [3.11–6.93]‰^2^ for adults. Juvenile SEAb was significantly smaller than both the subadult (*p*(SEAb_juv_ > SEAb_sub_) < 0.001) and adult SEAb (*p*(SEAb_juv_ > SEAb_ad_) < 0.001) and there was no significant difference in SEAb between adults and subadults (*p*(SEAb_ad_ > SEAb_sub_) = 0.322). There was an estimated 0.35‰^2^ of overlap between juveniles and subadults, corresponding to 26% and 6% of their SEAb, respectively; 0.67‰^2^ of overlap between juveniles and adults, corresponding to 49% and 13%, respectively; and 3.56‰^2^ of overlap between subadults and adults, corresponding to 61% and 70%, respectively.

### 3.2 Seasonal Resource Use

**FIGURE 5:**
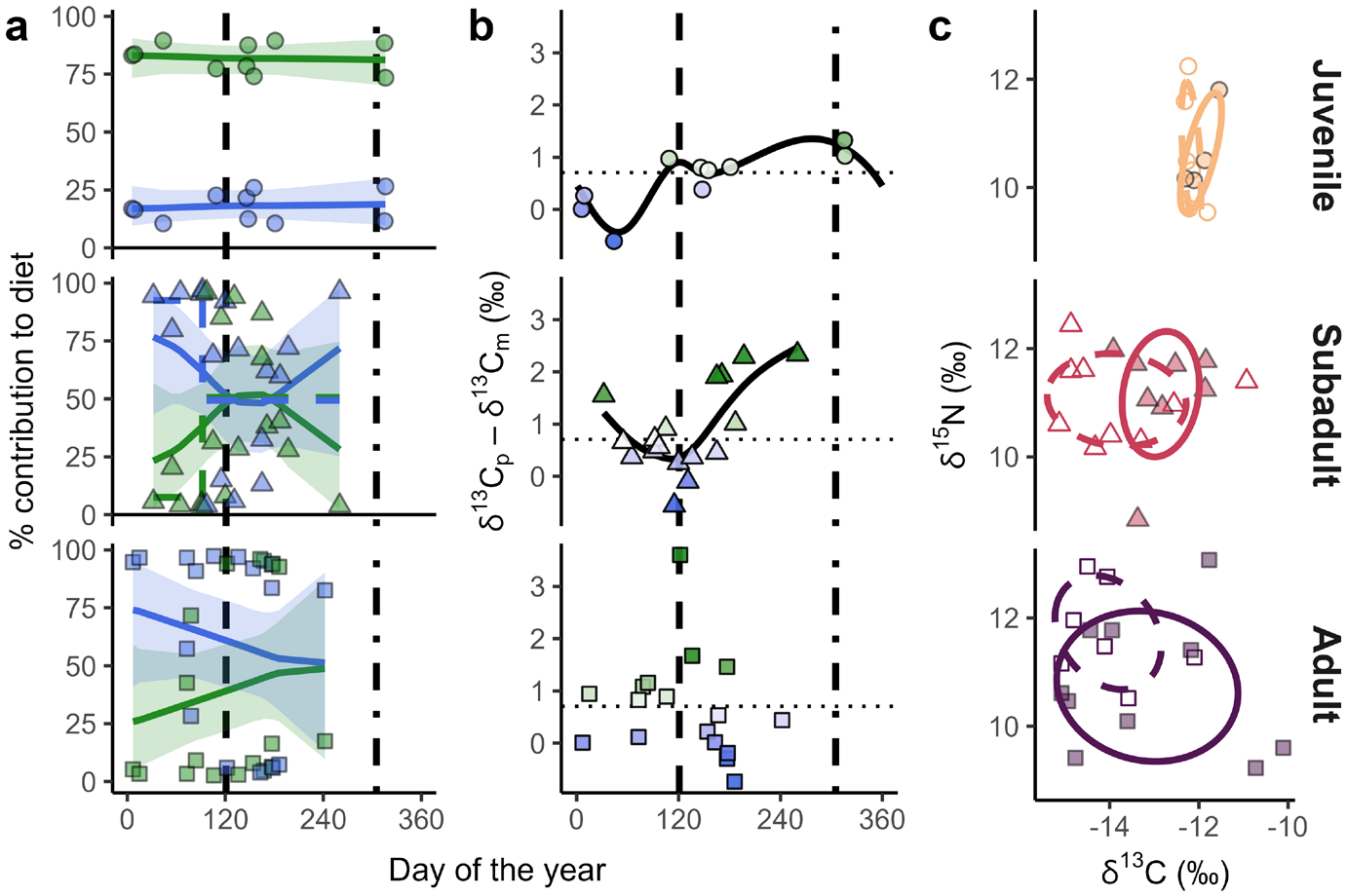
(a) Post hoc models of individual dietary contributions (points) predicted by the mixing model, with habitat contributions depicted by DOY for each age class. Solid lines correspond to fitted Bayesian Dirichlet regressions, with 95% credible interval ribbon. Dashed lines represent significant breakpoints and the means before and after. Vertical dashed and dot‐dash lines represent the start of the wet and dry seasons, respectively. (b) The inter‐tissue difference in *δ* ^13^C by DOY, where solid lines correspond to significant generalized additive models. The horizontal dotted line represents the expected inter‐tissue difference of 0.70‰. Green indicates positive inter‐tissue difference relative to expected, and blue indicates negative, interpretated as short‐term increased use of bay and coastal resources, respectively, with stronger colors indicating greater deviations from the expected value. (c) Standard ellipse areas from plasma values by season for each age class, where dashed ellipse and hollow points correspond to the dry season and solid lines and solid points correspond to the wet season.

Dirichlet regressions of habitat contributions against DOY by age class successfully converged according to R‐hat values of 1 and ESS values > 400 (Vehtari et al. 2021). Fitted smooth curves and their 95% credible intervals are displayed in Figure 5a. Bay resource use was predicted to remain high year‐round in juveniles, and correspondingly, no breakpoints were detected. In subadults, bay resource use was modeled to increase substantially around the start of the wet season, further supported by a significant breakpoint detected at DOY 92 corresponding to the beginning of April, ~1 month before the start of wet season on May 1^st^ (DOY 121). The regression model also predicted a return shift to coastal resource use in the late wet season, but there were insufficient individuals for a second breakpoint to be detected. In adults, bay resource use was modeled to modestly increase from the late dry season into the wet season, but no breakpoints were detected.

There was a significant non‐linear cyclic effect of DOY on the inter‐tissue difference in *δ* ^13^C in juveniles (edf = 4.44, *F* = 3.45, *p* = 0.023), explaining 84.3% of the deviance (adjusted *R* ^2^ = 0.717) and suggesting an inter‐tissue difference largely consistent with the expected value year‐round, except for a slight dip in the late dry season. There was also a significant non‐linear effect of DOY on the inter‐tissue difference in subadults (edf = 3.14, *F* = 5.61, *p* = 0.009), explaining 64.5% of the deviance (adjusted *R* ^2^ = 0.558) and suggesting a significant increase in the inter‐tissue difference going into the wet season. No significant non‐linear effect of DOY on the inter‐tissue difference was observed for adults (edf = 4.36, *F* = 2.05, *p* = 0.139).

Using plasma isotope values, SEAb for the dry and wet seasons, respectively, were 0.65 [0.15–1.26]‰^2^ and 0.77 [0.12–1.73]‰^2^ for juveniles, 3.67 [1.33–6.30]‰^2^ and 2.67 [0.91–4.77]‰^2^ for subadults, and 3.13 [0.96–5.72]‰^2^ and 7.88 [2.92–12.92]‰^2^ for adults. There was no significant difference between the SEAb of seasons for either juveniles (*p*(SEAb_dry_ > SEAb_wet_) = 0.446) or subadults (*p*(SEAb_wet_ > SEAb_dry_) = 0.250), but in adults, the dry season SEAb was significantly smaller than that of the wet season (*p*(SEAb_dry_ > SEAb_wet_) = 0.030). Dry and wet season SEAb had 0.07‰^2^ of overlap in juveniles (5% of the total area of both seasons), 1.00‰^2^ (19%) in subadults, and 1.50‰^2^ (16%) in adults.

## 4 Discussion

### 4.1 Methodological Considerations

Prior to the discussion of our findings, there are two primary methodological concerns that influenced decisions about analysis for this study and merit discussion. The first is that there are inherent challenges to studying the isotopic signatures of very young sharks, which may be affected by inputs from their mothers and therefore fail to accurately reflect only juvenile diet (Niella et al. 2021; Olin et al. 2011). In many shark species, including the great hammerhead, gestating females nourish developing embryos via a placental connection and provision pups with energy reserves in the form of an enlarged liver, on which neonate sharks will initially depend following parturition (Hussey, Wintner, et al. 2010). Consequently, the isotopic signature of the mother plus potential maternal‐fetal enrichment (Vaudo et al. 2010) lingers in her pups for a period after birth, which can bias measured isotopes of juvenile sharks (Niella et al. 2021; Olin et al. 2011). The isotopic dilution of maternal influence remains poorly understood in most species, leading many studies to omit sampled individuals less than one year old from analyses investigating resource use over ontogeny to minimize potential maternal bias (e.g., Bond et al. 2018).

Maternal influence has been proposed to last potentially up to 3.5 years (Niella et al. 2021), but we find this improbable in great hammerheads, which may have a growth rate of over 50 cm yr^−1^ in their first years (Macdonald et al. 2021) and will have roughly tripled in size by the time they are 3.5 years old (Piercy et al. 2010). Additionally, Lin et al. (2024) recently published a study on neonate and juvenile great hammerheads in the Arabian Gulf, which showed that by ~80–90 cm TL (corresponding to an FL of ~60–70 cm; Hsu et al. 2021), the muscle *δ* ^15^N values had declined from maternally enriched values to values within the range maintained throughout the remainder of the size range of juveniles sampled (maximum: 236 cm TL). Therefore, given the recent evidence for a rapid dilution of maternal influence in great hammerhead muscle and the extreme somatic growth exhibited by great hammerhead neonates, we interpreted the muscle values of the small sharks sampled in this study as indicative of their feeding ecology, except for the smallest two individuals (63 and 67 cm FL). While the *δ* ^15^N values of these individuals did not show obvious maternal enrichment relative to older juveniles, the *δ* ^13^C values of both their tissues were lower than the other individuals sampled at the nursery and were more comparable to those of many adults. Owing to the risk of maternal influence, we omitted these two individuals from statistical analyses. It is worth noting, however, that if maternal influence was indeed sufficiently diluted even in these individuals such that their isotopes reflect their foraging, their *δ* ^13^C values would suggest that pupping (estimated size‐at‐birth is ~50 cm FL; Piercy et al. 2010) may have occurred offshore, after which these individuals migrated to the nursery, arriving only shortly prior to being sampled based on the low *δ* ^13^C values still seen in their plasma.

The second methodological consideration is that estimating the relative contribution of various prey sources to the diet of great hammerheads required an approximate diet‐tissue discrimination factor to be supplied to the Bayesian mixing model. As DTDFs have not previously been estimated for the tissues of great hammerheads or their close relatives, we chose to use the DTDF of muscle estimated from other large‐bodied apex predatory shark species (Sand tiger *Carcharias taurus* and lemon sharks *Negaprion brevirostris* ; Hussey, Brush, et al. 2010) rather than apply published DTDFs for an ecologically dissimilar species (the leopard shark) since not only is DTDF shown to be diet dependent (Caut et al. 2013; Kim, Del Rio, et al. 2012) but our methodological preparation of plasma samples differed from Kim, Del Rio, et al. (2012) who did not perform lipid‐ or urea‐extractions on their plasma as we opted to do, which can have significant effects on plasma isotope ratios (Crook et al. 2019). This decision first required plasma to be corrected to the muscle values using the mean differences for each isotope (Raoult et al. 2019), which rests on the assumption that the muscle and plasma values are correlated 1:1 when in equilibrium with diet such that residuals reflect short‐term shifts in diet (Matich and Heithaus 2014). We tested this assumption in our data in the same manner used by Matich and Heithaus (2014) to validate their use of the mean difference between bull shark ( *Carcharhinus leucas* ) blood and plasma. The mean difference values calculated here were comparable to the published differences between extracted muscle and plasma of other elasmobranch species (Crook et al. 2019).

### 4.2 Ontogenetic and Seasonal Shifts in Resource Use

Using multi‐tissue stable isotope analysis, we present the first characterization of resource use and isotopic niche size across the entire ontogenetic spectrum of great hammerheads in the Western Atlantic. We found that resource use shifts from bay to coastal prey with growth, such that individuals appear to closely associate with bay nursery habitats until ~125 cm FL (or ~2 years old; Piercy et al. 2010), correspondingly classified here as ‘juveniles.’ Both larger immature ‘subadults’ and mature ‘adults’ exhibited mixed use of bay and coastal resources across the remainder of the size spectrum sampled, suggesting a partial ontogenetic shift where some continued use of inshore resources is expected in the older age classes.

Overall, the great hammerheads sampled in this study produced a range of muscle *δ* ^13^C values consistent with inshore estuarine (*c*. –10‰ to −14‰; Brownscombe et al. 2022; Chasar et al. 2005; James et al. 2022; Kieckbusch et al. 2004) or coastal reef foraging habitats (*c*. –14‰ to −17.5‰; Blanar et al. 2021), with little to no evidence of substantial pelagic foraging (*c*. –17‰ to −18.5‰; Moore 2014; Weidner et al. 2017). These values corroborate the findings of horizontal and vertical movement studies of great hammerheads, which indicate that core habitat use areas largely fall in coastal Florida state waters (Graham et al. 2016) with a preference for depths of < 30 m (Guttridge et al. 2022). Despite their much larger maximum size and reputation for preying on other sharks (De Bruyn et al. 2021; Gallagher and Klimley 2018; Lubitz et al. 2023; Mourier et al. 2013; Raoult et al. 2019; Roemer et al. 2016), when compared to the southern stingray and Atlantic sharpnose shark, mesopredators presented here to represent diet endmembers, the observed muscle *δ* ^15^N values of great hammerheads were only modestly higher, suggesting that they are predominantly tertiary consumers across ontogeny, with some individuals engaging in higher trophic level feeding. For the great hammerheads of Australia, Raoult et al. (2019) presented evidence of a more pronounced ontogenetic increase in trophic position and little evidence of switching food webs across ontogeny, although size was not a linear predictor of trophic level and food web dependence varied substantially among individuals. Similarly, we found that fork length alone was an ineffective linear predictor of raw isotope values due to substantial variation among individuals as size increased.

Bay batoids were predicted to contribute the most to the diet of juvenile great hammerheads. Surprisingly, their contribution declined with ontogeny as the importance of coastal teleost prey increased. A subset of subadults and adults, however, were modeled to still rely heavily on bay batoids, indicating the probable importance of rays across ontogeny. Stomach content analysis of Australian great hammerheads suggested that they feed preferentially on rays while also consuming teleost prey (De Bruyn et al. 2021), a finding that stable isotope models elsewhere appear to corroborate (Lubitz et al. 2023; Raoult et al. 2019). Interestingly, the apparent switch from a ray‐ to a teleost‐dominated diet observed here is the reverse of what Hsu et al. (2022) found in juvenile great hammerheads of the Arabian Gulf, which shifted from specializing on bartail flathead ( *Platycephalus indicus* ) to substantial exploitation of batoid prey by the age of 3 (or ~150 cm FL in the Atlantic population; Piercy et al. 2010). The predicted importance of rays especially to the diet of Biscayne Bay's juvenile great hammerheads might also suggest that Biscayne Bay's abundance of small shallow‐water ray species—e.g., large populations of yellow stingrays ( *Urobatis jamaicensis* ; Francis 2023) which even neonate great hammerheads may be capable of targeting—may help explain the value of the area as a nursery habitat.

Great hammerheads are also known to target other large sharks, with this behavior having been directly observed (Mourier et al. 2013; Roemer et al. 2016) as well as predicted in the isotope mixing model of Raoult et al. (2019) who estimated a substantial relative contribution from carcharhinid sharks in addition to rays. Aside from a few individuals, however, coastal sharks—represented here by the small Atlantic sharpnose shark (*R. terraenovae*)—were modeled to be a relatively minor contributor to the diet of great hammerheads across ontogeny, perhaps due to small sharks' use of sheltered habitats or schooling behavior (Branstetter 1990; Heithaus 2007). Others have suggested that sharks are not a major dietary component of great hammerheads (Mourier et al. 2013), which our model appears to corroborate. Smaller sharks are likely a high‐cost, high‐reward prey item for great hammerheads, with rays and teleosts making greater dietary contributions (De Bruyn et al. 2021) potentially due to their greater availability and/or lower energetic cost. To the best of our knowledge, the consumption of other sharks is not a behavior that has been documented in small immature great hammerheads to date, a precedent that we believe our model currently supports despite abundant small sharks in Biscayne Bay—namely, neonate blacktip sharks ( *C. limbatus* ), neonate nurse sharks ( *Ginglymostoma cirratum* ), and bonnethead sharks ( *S. tiburo* ) in addition to sharpnose sharks—that may be suitably sized prey even for a small great hammerhead during nursery association. Several of our subadults, however, were modeled to have coastal shark diet contribution of ~50% indicating that they may begin to exploit this prey pool before reaching sexual maturity. Where subadult hammerheads consume coastal sharks, such consumption is likely to be opportunistic, as suggested by observations of successful or attempted depredation on hooked small sharks. For instance, in April 2025, a 153‐cm FL subadult was captured having depredated a hooked bonnethead shark and on other occasions, subadults have been observed following hooked small sharks including a blacktip shark, a blacknose shark (*C. acronotus*) and a juvenile tiger shark (*Galeocerdo cuvier*).

Overall, a diversity of trophic strategies was apparent in subadults and adults, some of which were modeled to have diets dominated by bay batoids, bay teleosts, or coastal sharks in addition to the majority heavily exploiting coastal teleosts. This diversity can be seen clearly in the significantly larger isotopic niches of these age classes compared to juveniles. While this is the first study of its kind on great hammerheads, niche expansion has been observed in scalloped hammerheads in the Galapagos Marine Reserve associated with shifts to incorporate offshore habitat use in subadults (Cerutti‐Pereyra et al. 2022), and smaller, more specialized niche sizes have similarly been documented in juvenile smooth and scalloped hammerheads in the Gulf of California (Besnard et al. 2024). Niche contraction in scalloped hammerheads elsewhere (Estupiñán‐Montaño, Galván‐Magaña, et al. 2021; Estupiñán‐Montaño, Tamburin, et al. 2021), however, reaffirms that sharks may display population‐dependent heterogeneity in the direction and extent of ontogenetic niche shifts.

Visualization of isotopic niches generated from raw muscle isotope values showed that the great hammerhead niche expands into isotopic space corresponding to foraging at higher trophic levels and in coastal reef habitats, without abandoning inshore and lower trophic‐level niche space. A similar ontogenetic niche expansion has also been observed in tiger sharks, corresponding to increased use of deeper environments without eliminating their use of surface waters, which was hypothesized to be associated with increasingly generalist feeding (Afonso and Hazin 2015). The observed niche expansion in great hammerheads may therefore indicate a shift from relative specialist to generalist feeding, exploiting a limited range of inshore bay resources at smaller sizes and incorporating deeper coastal resources as they become more accessible and predation risk declines. Modeled diets, however, also highlight the possibility that the large isotopic niche is the result of diverging specialization among individuals within the subadult and adult age classes, with at least a few individuals predicted to rely heavily on each bay and coastal resource group. Since the model used both muscle and plasma values, very high contribution estimates suggest trophic consistency between the two timescales integrated by the tissues. Using a similar multi‐tissue SIA approach, bull sharks have also been documented forming a generalist population composed of individual specialists (Matich et al. 2011).

The high degree of isotopic niche overlap between age classes and common prey groups suggests the possibility of shared resources between conspecifics of different sizes, which may ultimately lead to competition if resources become limited (Kinney et al. 2011; Shiffman et al. 2019) or even to direct predation (Guttridge et al. 2012). In the case of shared resources, competitive exclusion or high predation risk may restrict the foraging opportunities and niche space of nursery‐associated juveniles (Bullock et al. 2024; Kinney et al. 2011; Weideli et al. 2023) driving them to maintain a close association to the bay nursery to avoid predation.

Risks of predation and competition may also motivate subadults to shift foraging habitat seasonally. While the observed seasonal shifts in subadult great hammerhead habitat use may be driven by conditions like sea surface temperature and primary productivity as seen in juvenile smooth hammerheads (Logan et al. 2020) or prey pulses as seen in juvenile bull sharks (Matich and Heithaus 2014), given the probability for shared resource use between conspecifics, we propose that the seasonal shift detected in subadults may represent a strategy to minimize the risks of intraspecific competition or predation by temporally partitioning coastal foraging habitats (Guttridge et al. 2012; Kinney et al. 2011). Namely, catch data from this study allows some inferences about the seasonal abundance of adult great hammerheads in the study area, with ~80% of the adult catch density occurring from ~March–July, after which they either shift into deeper waters beyond the current extent of our coastal survey efforts or migrate to other locations. This hypothesis is supported by a recent report that mature great hammerheads in South Florida use offshore habitats during the summer and winter but shift into shallower nearshore habitats in the spring (Casselberry et al. 2025), which aligns with the observed modest increase in bay resource use during the wet season modeled in our adults. Additionally, Guttridge et al. (2017) found that large great hammerheads are highly resident to the habitats near Jupiter, FL (~150 km north of Biscayne Bay) and Bimini, The Bahamas (~85 km to the east) from November through April, constituting nearby habitats to which adults may migrate in the late summer to reside before returning the following spring. Intraspecific predation risk has been shown to drive juvenile shark habitat use of nursery‐associated lemon sharks, who tidally shift between foraging and taking shelter due to tidal variations in intraspecific predation risk (Bullock et al. 2024; Guttridge et al. 2012). Therefore, movements to avoid interactions with conspecifics may also be occurring in subadult great hammerheads, investigated here at larger spatial and temporal scales.

The seasonal shift in subadult resource use was detected in the post hoc analyses of habitat contributions to diet, but because the initial mixing model used both long‐term muscle and short‐term plasma to generate these estimates, it was important to rigorously confirm whether the observed seasonality was the result of a true foraging shift and not simply the detection of differences between individual specialists. By modeling the inter‐tissue difference, we quantified an individual's short‐term resource use relative to its average resource use (Matich and Heithaus 2014) and showed that subadult plasma becomes increasingly higher in *δ* ^13^C relative to muscle during the wet season, suggesting true changes in foraging habitat at the individual level. Finally, using raw plasma values, we found that the seasonal isotopic niche of subadults shifts substantially into more inshore space in the wet season, without significantly changing size and with only 19% of the total isotopic niche occupied during both seasons. Again consistent with the habitat shift reported by Casselberry et al. (2025), the adult isotopic niche also shifted noticeably with season, increasing significantly in size in the wet season by expanding further into more inshore and lower trophic position space, with only 16% of the total isotopic niche occupied during both seasons; however, it is important to note that there was no significant relationship with DOY detected in adult inter‐tissue difference, meaning that differences between individual specialists cannot be ruled out as the cause of the observed increases in bay resource use and inshore isotopic space during the wet season. While only 5% of the total isotopic niche of juveniles was occupied in both seasons, this was likely an artifact of their small seasonal niches, as the ellipses are visibly very similar and we observed no notable seasonal variation in foraging habitat from their modeled diets or inter‐tissue differences, indicative of year‐round nursery association in the first 2 years of a great hammerhead's life.

### 4.3 Conservation Implications

The smaller, potentially more specialized isotopic niche and year‐round nursery dependence of juveniles, combined with the possibility of intraspecific competition and predation into subadulthood, lead us to conclude that immature great hammerheads likely face constraints to resource availability and foraging opportunities in early life, increasing their vulnerability to anthropogenic disturbances in an already highly human‐impacted habitat (Carbonell et al. 2021; De Sousa Rangel et al. 2021; Macdonald et al. 2021; Munroe et al. 2014; Santos et al. 2020). In addition to risks associated with habitat loss and degradation, great hammerheads in areas surrounding the nursery—which notably include gestating females in addition to immature individuals—experience threats from recreational fishing; the South Florida coast, including Biscayne Bay, is a recreational fishing hot spot (Casselberry et al. 2024; Lerner et al. 2017; Stoa 2016), putting great hammerheads at risk of capture‐related stress, injuries, and mortality (Binstock et al. 2023; Ellis et al. 2017; Gulak et al. 2015; Jerome et al. 2018). In May 2024, we recovered and sampled a dead 157‐cm FL (~3.5 year old; Piercy et al. 2010) subadult male that had washed up on a beach ~5.6 km north of the nursery site. It was still bleeding from a hooking injury to its lower jaw, providing unequivocal evidence of recent post‐release mortality.

Fishing pressures and declines in habitat quality pose not only a direct threat to juvenile great hammerheads but might also jeopardize access to key prey species (Kellison et al. 2012), emphasizing the importance of continuing to identify and manage important prey taxa using higher resolution minimally invasive methods like fecal DNA metabarcoding (Van Zinnicq Bergmann et al. 2021). The use of coastal foraging habitats and prey by immature great hammerheads outside of the identified nursery site highlights the need for local movement studies to continue to refine the spatial mosaic of habitats that fall within the seascape nursery (Nagelkerken et al. 2015). To build on this research and facilitate the conservation of great hammerheads and management of the local nursery, we suggest that future studies aim to (1) more precisely track the fine‐scale movements of, and habitats used by, young great hammerheads across seasons and ontogeny, (2) identify key prey taxa they exploit during nursery association, and (3) determine to what extent young great hammerheads may compete for resources within the broader predatory fish community of South Florida.

This investigation of great hammerhead trophic ecology around a South Florida nursery provides evidence of constrained or specialized foraging in great hammerheads associated with year‐round dependence on bay nursery resources during their first two years of life. Older subadults expand into coastal foraging habitats but exhibit seasonal increases in bay foraging in the wet season, possibly to avoid interactions with more abundant adult conspecifics during those months. Importantly, our findings reaffirm that shifts in resource use can vary among hammerhead species and across populations, emphasizing the importance of species‐, population‐, and ontogeny‐specific investigations into trophic ecology across seasons to inform local management (Cerutti‐Pereyra et al. 2022; Estupiñán‐Montaño, Galván‐Magaña, et al. 2021; Estupiñán‐Montaño, Tamburin, et al. 2021; Raoult et al. 2019). This study provides both the necessary foundation and motivation for future investigations to further resolve diet and habitat use of local great hammerheads for targeted management of this critical nursery for the Western Atlantic population.

## Author Contributions

**John F. Hlavin:** conceptualization (equal), data curation (equal), formal analysis (lead), funding acquisition (supporting), investigation (equal), visualization (lead), writing – original draft (lead), writing – review and editing (equal). **Catherine C. Macdonald:** conceptualization (equal), data curation (equal), funding acquisition (lead), investigation (equal), resources (lead), supervision (lead), writing – review and editing (equal).

## Conflicts of Interest

The authors declare no conflicts of interest.

## Data Availability

All data and code that support the findings of this study are openly available on Figshare at the following DOI: https://doi.org/10.6084/m9.figshare.26082184.

## Appendix 1 Sample Sizes (*n*), Average and Range of Fork Lengths (cm), Average and Range of *δ* ^13^C (‰), and Average and Range of *δ* ^15^N (‰) of Sampled Great Hammerhead Muscle and Plasma, Broken Down by Sex, Maturity, Size Class, and Season (Plasma)

**Table:**

	Demographic	*n*	Mean FL (cm)	FL Range (cm)	Mean *δ* ^13^C (‰)	*δ* ^13^C Range (‰)	Mean *δ* ^15^N (‰)	*δ* ^15^N Range (‰)
Muscle	All	62	187 (± 60)	63–287	−13.7 (± 1.5)	−16.3: −10.7	13.2 (± 1.2)	9.1–15.5
	F	38	191 (± 61)	67–287	−13.8 (± 1.5)	−16.3: −10.7	13.2 (± 1.3)	9.1–15.5
	M	24	182 (± 58)	63–259	−13.6 (± 1.5)	−16.2: −10.8	13.2 (± 1.0)	10.6–15.0
	Immature	34	146 (± 50)	63–222	−13.6 (± 1.3)	−16.3: −11.5	13.2 (± 1.1)	9.1–15.5
	Mature	28	237 (± 21)	187–287	−13.9 (± 1.7)	−16.2: −10.7	13.2 (± 1.2)	10.3–15.5
Plasma	All	46	175 (± 61)	63–264	−13.2 (± 1.3)	−15.1: −10.1	11.0 (± 1.0)	8.8–13.1
	F	2	178 (± 61)	67–264	−13.2 (± 1.3)	−15.1: −10.7	10.9 (± 1.0)	8.8–12.8
	M	17	171 (± 62)	63–240	−13.1 (± 1.4)	−15.1: −10.1	11.1 (± 1.1)	9.5–13.1
	Immature	29	143 (± 52)	63–222	−13.0 (± 1.2)	−15.1: −10.9	10.9 (± 0.9)	8.8–12.4
	Mature	17	231 (± 17)	187–264	−13.5 (± 1.6)	−15.1: −10.1	11.1 (± 1.2)	9.2–13.1
	Dry	24	164 (± 65)	63–264	−13.5 (± 1.3)	−15.1: −10.9	11.1 (± 1.0)	9.5–13.0
	Wet	22	188 (± 54)	85–262	−12.8 (± 1.4)	−15.1: −10.1	10.9 (± 1.1)	8.8–13.1
